# Parliament and the Profession

**Published:** 1916-01-29

**Authors:** 


### PARLIAMENT AND THE PROFESSION.

### Patriotism of the Medical Profession.

Replying to Mr. Lynch, Mr. Tennant pointed out that machinery is in existence, in the shape of the Central Medical War Committee, to advise as to the best use which can be made of medical men presenting themselves for acceptance as candidates for commissions in the R.A.M.C. Thanks to the patriotism of the medical profession, there is no present anxiety regarding the supply of doctors for the Army.

### Enteric Fever in the Army.

Mr. W. Thorne asked the Under-Secretary for War whether cases bearing the name of paratyphoid fever, trench fever, pyrexia, "and other pseudonyms of enteric fever" were included in the totals furnished by him (The Hospital, January 15, p. 354). Mr. Tennant replied that the diseases mentioned were not included in the figures, and he could not accept the suggestion that those diseases are pseudonyms for enteric fever. He repudiated the suggestion that the medical authorities hide cases of enteric fever by falsely returning them under other names.

### Doctors and the Military Service Bill.

In Committee on the Military Service Bill, Mr. King moved an amendment to include among the exemptions fully qualified medical practitioners, registered dentists, and students at any hospital in their fourth and fifth years. Mr. Long, in the course of his speech opposing the amendment, paid a high tribute to the medical profession, but urged that to include in an automatic exemption all medical men and dentists would be a very dangerous proceeding. The amendment was negatived without a division. An amendment to exempt all medical students was withdrawn.

### Nerve Shock among Soldiers.

The treatment of nerve shock occurring among soldiers was again referred to by Mr. Hodge, who desired that the practice of placing the rank and file when so invalided under lunacy administration in annexes of county asylums should be put an end to. Mr. Tennant pointed out, however, that the institutions in question are entirely under the control of the War Office, and not under the jurisdiction of the Lunacy Board. The institutions that are being used in whole or in part for the treatment of these cases are Springfield War Hospital, Wandsworth; the Napsbury War Hospital, St. Albans; the Red Cross Military Hospital, Maghull, near Liverpool; and the War Hospital, Peebles.

### Doctors' Cars and Lighting Restrictions.

A question asked by Mr. Perkins suggested that medical men living in country districts, who frequently have to drive themselves long distances after dark in order to visit their patients, were greatly inconvenienced because they were required to use a minimum of light. The Home Secretary, however, said there must be a misunderstanding as to the effect of the Lighting Order. The use of headlights is prohibited in certain areas, but the limits of size and power for side-lights have been fixed sufficiently high to allow a good driving-light for doctors and other motorists who have to use country roads at night.

### Other Matters of Interest.

The Pay of Pensioned Medical Officers.—With the exception of a small number of surgeon-generals who have given their services in posts of a grading not commensurate with that rank, retired officers of the R.A.M.C. who have been called up for active service are now receiving full pay of rank in addition to full pension.

Artificial Limbs for Soldiers.—Replying to Sir George Toulmin, Mr. Tennant stated that officers who have lost limbs are given such sums as the Army Council consider sufficient to defray the necessary expense of providing artificial appliances, and artificial limbs for men are provided and prepared at the public cost for men who lose limbs in the war.

The Pay of Dental Mechanics.—The pay received by dental mechanics who are in the R.A.M.C. is 1s. 6d. a day, whereas in private employment these men are paid £3 a week. In the view of Sir J. Harmood Banner this involves a hardship. Mr. Forster pointed out that in addition to pay the men received food, clothing, accommodation, and separation allowance for family and dependants.

Medical Examination of Recruits.—It is common knowledge that under the Derby recruiting scheme many men were only subjected to a cursory medical examination, and when they are called up they will therefore be put through a more searching examination, when those found unfit for service will be sent back to civil life. Answering Mr. Thomas, who pointed out that this would cause loss and inconvenience to men who have to dispose of their business, Mr. Tennant said that every effort would be made to enable the men to be examined before they sever their connection with civil occupation.

Army Recruits and Eyesight Tests.—Major Lane-Fox suggested that many otherwise suitable men are being rejected from enlistment on the ground of defective sight. Mr. Tennant, in his reply, said the minimum standard for eyesight for general service was low, and that soldiers were given glasses to bring their sight more nearly to the normal; there were, however, cases where even glasses would not bring the sight to a standard sufficient for general service. A mere statement of inadequacy of vision is not regarded as a sufficient reason for excusing from military service.

